# The association between parity and metabolic syndrome and its components in normal-weight postmenopausal women in China

**DOI:** 10.1186/s12902-020-00674-6

**Published:** 2021-01-07

**Authors:** Mengte Shi, Xinhe Zhou, Chao Zheng, Youjin Pan

**Affiliations:** 1 Department of Endocrinology, The Second Affiliated Hospital and Yuying Children’s Hospital of Wenzhou Medical University, No. 109, Xueyuanxi Road, Wenzhou, Zhejiang, 325000 China; 2 The Second Affiliated Hospital of Zhejiang University School of Medicine, Hangzhou, People’s Republic of China

**Keywords:** Parity, Normal weight, Metabolic syndrome, Postmenopausal

## Abstract

**Background:**

Studies analyzing the association between parity and normal-weight metabolic syndrome (MetS) in postmenopausal women of normal weight remain limited, this study aimed to explore the association between parity and MetS among Chinese normal-weight postmenopausal women.

**Methods:**

In total, 776 normal-weight undiagnosed type 2 diabetes postmenopausal women who visited the Second Affiliated Hospital and Yuying Children’s Hospital of Wenzhou Medical University for a routine health check-up between 1 January 2017 and 31 December 2019 were included in the cross-sectional study. All individuals had fully completed information records encompassing standardized electronic medical records, physical examinations, and biochemical measurements. Metabolic health was defined as fewer than 2 parameters of the MetS were present, in combination with normal weight. Continuous variables which were normally distributed were expressed as means and standard deviation. Comparisons among normally distributed continuous variables were made using one-way ANOVA while that among non-normal distribution parameters were made using Kruskal-Wallis. The association between parity and MetS was analyzed using multivariate logistic regression. All of the analyses were performed with SPSS statistical software (Version 23.0, SPSS, Inc., Chicago, IL, USA) and the statistical software package EmpowerStats (http://www.empowerstats.com, X&Y Solutions, Inc., Boston, MA).

**Results:**

After adjusting for potential confounding factors including hip circumference, parity was failed to show a significantly relationship with MetS in normal-weight women(*P*=0.054). Women with a higher parity (≥3) had an increased OR of abdominal obesity, while the OR (95% CI) of the parity 3 group was 2.06 (1.13, 3.77) and that of the parity ≥4 group was 3.08(1.42, 6.66) the P for trend was 0.002 after adjusting for potential confounding factors. No significant differences were detected for other metabolic disorders including high levels of triglycerides (TG), blood pressure, fasting plasma glucose (FPG), and decreased high-density lipoprotein cholesterol (HDL-C) in different parity groups.

**Conclusions:**

Higher parity was not associated with a higher risk of MetS in normal weight Chinese postmenopausal women. As for the components of MetS, only waist circumference was associated with multiparity even after controlling for hip circumference.

## Background

In recent years, the incidence of metabolic syndrome (MetS) has markedly increased in China due to the westernization of life. Thus far, epidemiologic studies have suggested that MetS prevalence in China was 9.8 to 18.2% in different districts according to different diagnostic criteria [1–5]. Studies have found that women had a higher prevalence in elderly groups than men after 60 years of age (47.9% vs 27.6) [5]. Compared to men, female reproductive factors may explain the disparity among the elderly. Therefore, researchers have been interested in studying the correlation between female reproductive factors and MetS.

Nowadays, information on the global prevalence of MetS among individuals of normal weight is scarce [6–8]. In China, Zhang et al. [6] reported that MetS prevalence was 8.14% among normal-weight individuals in Beijing; Zheng et al. concluded that the prevalence of metabolic abnormality was 34.1% in normal-weight individuals [7]. And several prospective studies have focused on the risk for cardiovascular disease (CVD) among normal-weight [a body mass index (BMI) of 18.5–24.9 kg/m^2^] metabolically unhealthy or metabolic abnormal individuals, although the definition of metabolically unhealthy or metabolic abnormal was inconsistent in these studies [9–11]. Previous studies [9, 12–15] have also indicated that the risk of CVD in metabolically unhealthy normal-weight individuals was about 1.5 to 3-fold higher than metabolically healthy individuals of normal weight. Therefore, it is necessary to focus on metabolic status in normal weight individuals.

Pregnancy is considered to be accompanied by major alterations in the metabolic system, which may play an important role in the metabolic abnormalities of postmenopausal women [16]. Although numerous studies have focused on the association between parity and MetS, the results have been inconsistent. To-date, studies analyzing the association between parity and MetS in normal weight postmenopausal women remain limited. Accordingly, this study aims to evaluate the associations between parity and MetS as well as its components among postmenopausal women of normal-weight.

## Methods

### Research subjects

In this cross-sectional study, a total of 1936 undiagnosed type 2 diabetes postmenopausal women aged 40 to 75 years old from Nan Pu and Pu Xie Shi communities of Wenzhou City were recruited to take a routine health check in Wenzhou Medical University Healthcare Center between 1 January 2017 and 31 December 2019. Participants with normal weight (BMI ≥18.5 and < 24) were enrolled. After excluding subjects with incomplete data, and following diseases: established cardiovascular and cerebrovascular diseases, severe liver or renal dysfunction, acute infection, tumors, and psychiatric disease, 776 undiagnosed type 2 diabetes postmenopausal women with normal weight were included in the original analysis. All participants completed their standardized electronic medical records, which included their demographic characteristics, reproductive history, lifestyle information such as physical activity, smoking and drinking status. This study was approved by the Institutional Review Board of the Second Affiliated Hospital of Wenzhou Medical University (KYKT2018–112).

### Physical examination and laboratory tests

Physical examinations including body height, weight, waist circumference (WC) and hip circumference along with blood pressure assessment were performed in the morning of testing a day after fasting for least 8 h. Body weight and height were measured with light clothing and without shoes. The BMI was calculated as weight (kg)/height (m)^2^. Blood pressure was measured by Omron® intelligence electronic blood pressure monitor with the patient supine after resting for 10 min. Laboratory tests included glucose metabolism indexes such as fasting plasma glucose (FPG), 2 h postprandial glucose (2hPG), and hemoglobin A1c (HbA1c). Undiagnosed diabetic participants took 75 g glucose orally, and lipid levels including total cholesterol (TC), triglyceride (TG), high-density lipoprotein cholesterol (HDL-C), low-density lipoprotein cholesterol (LDL-C) and liver functions were analyzed.

### Record of lifestyle information

The International Physical Activity Questionnaire was used to evaluate the physical activity by asking questions related to the frequency and duration of moderate and vigorous activities. Physically active was defined as having at least 150 min/week moderate-intensity activity, 75 min/week vigorous-intensity activity, or ≥150 min/week for a combination of the two [17]. Current smoking was defined as smoking at least one cigarette per day for > 6 months. Current drinking was defined as consuming one or more alcoholic drink on ≥1 day in a week during the past half year.

### Definition of normal-weight, MetS and metabolic health

Normal-weight was defined as a normal BMI (≥18.5 and < 24) in China [7]. MetS was diagnosed according to the criteria of the American Heart Association and the National Heart, Lung, and Blood Institute, together with the International Diabetes Federation in 2009: (1) WC of ≥80 cm for women, according to Asian-Pacific criteria; (2) fasting glucose of ≥100 mg/dl or current treatment for elevated glucose; (3) fasting triglycerides of ≥150 mg/dl or medication use; (4) HDL-C of < 50 mg/dl or medication use; and (5) systolic blood pressure (SBP) of ≥130 mmHg, diastolic blood pressure (DBP) of ≥85 mmHg or under drug treatment for hypertension [18]. Metabolic health was defined as fewer than 2 parameters of the MetS were present, in combination with normal weight [19].

### Statistical analysis

Continuous variables which were normally distributed were expressed as means and standard deviation while non-normal distribution parameters were given as medians and interquartile ranges (IQR). Comparisons among normally distributed continuous variables were made using one-way ANOVA while that among non-normal distribution parameters were made using Kruskal-Wallis. Additionally, categorical variables were analyzed by the Chi-square test. Statistical analyzes were performed using the SPSS statistical software (Version 23.0, SPSS, Inc., Chicago, IL, USA), and analyses of the relationship between parity and MetS were performed using the statistical software package EmpowerStats (http://www.empowerstats.com, X&Y Solutions, Inc., Boston, MA). *P* values less than 0.05 (two-sided) were considered to be statistically significant.

## Results

### Baseline characteristics

A total of 776 postmenopausal women of normal weight were enrolled in the present study. Overall, 288 (37.1%) of the study population had MetS. Moreover, women with higher parity were more likely to be older, and a significant statistical difference was present among WC, hip circumference, FPG, 2hPG, HbA1c, SBP, DBP with the increase in parity. The levels of HDL-C had a significant statistical difference in relation to the increase in parity, however, TC, TG and LDL-C did not demonstrate any statistical difference. Lifestyle, such as alcohol drinking status, exhibited a significant difference across parity groups, while smoking status, physical activity did not. In terms of reproductive factors, age at menarche and duration of reproductive years were different in different parity categories, with no statistical difference in age at menopause among different parity groups. More details are listed in Table 1.

**Table 1:** Baseline Characteristics of participants (*N* =776), by number of parity

Variable	Parity				*P*-value
	1	2	3	≥4	
N (%)	217(28.0)	283(36.5)	179(23.0)	97(12.5)	
Age, mean (SD)	54.40 ± 4.42	59.62 ± 5.60	63.21 ± 5.99	64.87 ± 5.97	< 0.001
WC (cm)	77.71 ± 6.17	80.17 ± 6.43	82.43 ± 7.52	83.73 ± 7.92	< 0.001
Hip circumference (cm)	90.12 ± 4.72	91.67 ± 5.10	93.85 ± 7.07	95.09 ± 7.86	< 0.001
BMI (kg/m^2^)	21.65 ± 1.62	22.00 ± 1.41	21.95 ± 1.46	22.02 ± 1.50	0.118
FPG (mmol/L)	5.45 ± 1.01	5.50 ± 0.79	5.54 ± 0.78	5.55 ± 0.77	0.024
2hPG (mmol/L)	6.85 ± 2.69	7.33 ± 2.46	7.77 ± 2.57	8.32 ± 3.09	< 0.001
HbA1C (%)	5.76 ± 0.56	5.80 ± 0.51	5.85 ± 0.47	5.85 ± 0.48	0.017
HDL (mmol/L)	1.58 ± 0.34	1.47 ± 0.33	1.50 ± 0.33	1.48 ± 0.30	0.006
LDL (mmol/L)	3.4 ± 0.9	3.3 ± 0.9	3.3 ± 0.8	3.3 ± 0.8	0.548
TC (mmol/L)	5.79 ± 1.00	5.66 ± 1.05	5.63 ± 1.01	5.68 ± 0.99	0.340
TG (mmol/L)	1.50 ± 0.81	1.69 ± 1.12	1.66 ± 1.05	1.67 ± 0.89	0.131
SBP (mmHg)	123.51 ± 15.89	129.12 ± 17.71	134.34 ± 17.60	137.61 ± 17.89	< 0.001
DBP (mmHg)	76.47 ± 10.01	76.56 ± 9.76	77.97 ± 9.72	79.36 ± 9.89	0.036
Age at menarche (y)	15.68 ± 1.77	16.13 ± 1.72	16.47 ± 1.78	17.03 ± 1.96	< 0.001
Age at menopause (y)	49.31 ± 4.09	50.04 ± 3.87	49.77 ± 3.91	49.28 ± 5.07	0.269
Duration of reproductive years (y)	33.64 ± 4.41	33.91 ± 4.03	33.30 ± 4.29	32.25 ± 5.31	0.033
Alcohol drinker, n (%)					0.002
Never (548)	129 (59.4%)	213 (75.3%)	133 (74.3%)	73 (75.3%)	
Current (220)	83 (38.2%)	69 (24.4%)	44 (24.6%)	24 (24.7%)	
Former (8)	5 (2.3%)	1 (0.4%)	2 (1.1%)	0 (0.0%)	
Smoking, n (%)					0.868
Never	216 (99.54%)	281 (99.29%)	178 (99.44%)	97 (100.00%)	
Current	1 (0.46%)	2 (0.71%)	1 (0.56%)	0 (0.00%)	
Physical activity					0.201
yes	52 (23.96%)	70 (24.73%)	41 (22.91%)	14 (14.43%)	
no	165 (76.04%)	213 (75.27%)	138 (77.09%)	83 (85.57%)	
Educational attainment					< 0.001
Illiteracy	9 (4.15%)	35 (12.37%)	56 (31.28%)	37 (38.14%)	
Primary school	56 (25.81%)	101 (35.69%)	73 (40.78%)	43 (44.33%)	
Middle school	108 (49.77%)	103 (36.40%)	38 (21.23%)	15 (15.46%)	
High school	41 (18.89%)	37 (13.07%)	11 (6.15%)	2 (2.06%)	
College or above	3 (1.38%)	7 (2.47%)	1 (0.56%)	0 (0.00%)	
FDR					< 0.001
Yes	175 (80.65%)	255 (90.11%)	163 (91.06%)	94 (96.91%)	
No	42 (19.35%)	28 (9.89%)	16 (8.94%)	3 (3.09%)	
MetS					< 0.001
No	166 (76.5%)	180 (63.6%)	93 (52.0%)	49 (50.5%)	
Yes	51 (23.5%)	103 (36.4%)	86 (48.0%)	48 (49.5%)	

Values are mean ± SD

Abbreviations: *WC* Waist circumference, *BMI* Body mass index, *HbA1c* Hemoglobin A1c, *FPG* Fasting plasma glucose, *2hPG* 2 h postprandial glucose, *TC* Total cholesterol, *TG* Triglyceride, *HDL-C* High density lipoprotein cholesterol, *LDL-C* Low density lipoprotein cholesterol, *SBP* Systolic blood pressure, *DBP* Diastolic blood pressure, *FDR* First-degree relatives of patients with diabetes, *MetS* Metabolic syndrome

### Univariate analysis and multivariate logistic regression analysis

The results of the univariate analysis demonstrated that age, SBP, DBP, FPG, BMI, WC, hip circumference, HDL-C, TG, parity and educational attainment were correlated with MetS in normal-weight women (Table 2).

**Table 2:** Univariate analysis for MetS in normal weight individuals

Variable	Statistics	OR (95%CI)	*P-*value
Age, mean (y, SD)	59.64 ± 6.60	1.07 (1.04, 1.09)	< 0.001
WC (cm)	80.48 ± 7.16	1.17 (1.13, 1.20)	< 0.001
Hip circumference (cm)	92.17 ± 6.15	1.10 (1.07, 1.12)	< 0.001
BMI (kg/m^2^)	21.89 ± 1.50	1.53 (1.37, 1.71)	< 0.001
FPG (mmol/L)	5.50 ± 0.85	2.94 (2.29, 3.79)	< 0.001
HDL (mmol/L)	1.51 ± 0.33	0.03 (0.02, 0.06)	< 0.001
TG (mmol/L)	1.63±1.00	3.94 (3.06, 5.08)	< 0.001
DBP (mmHg)	77.21 ± 9.87	1.07 (1.05, 1.08)	< 0.001
SBP (mmHg)	129.82 ± 17.86	1.04 (1.03, 1.05)	< 0.001
Age at menarche (y)	16.20 ± 1.83	1.01 (0.93, 1.09)	0.846
Age at menopause (y)	49.68 ± 4.11	1.01 (0.97, 1.04)	0.772
Duration of reproductive years (y)	33.49 ± 4.39	1.00 (0.97, 1.04)	0.849
Alcohol drinker, n (%)			0.451
Never	548 (70.62%)	1.0	
Current	220 (28.35%)	0.81 (0.58, 1.12)	
Former	8 (1.03%)	0.96 (0.23, 4.05)	
Smoking, n (%)			0.620
Never	772 (99.5%)	1.0	
Current	4 (0.5%)	0.56 (0.06, 5.44)	
FDR			0.348
No	687 (88.5%)	1.0	
Yes	89 (11.5%)	0.80 (0.50, 1.28)	
Physical activity			0.765
No	599 (77.2%)	1.0	
Yes	177 (22.8%)	0.95 (0.67, 1.34)	
pregnancy losses			0.121
0	230 (29.6%)	1.0	
1	287 (37.0%)	0.67 (0.47, 0.96)	
2	166 (21.4%)	0.79 (0.52, 1.19)	
≥3	93 (12.0%)	1.01 (0.62, 1.64)	
Parity			< 0.001
1	217 (28.0%)	1.0	
2	283 (36.5%)	1.86 (1.25, 2.77)	
3	179 (23.1%)	3.01 (1.96, 4.62)	
≥4	97 (12.5%)	3.19 (1.92, 5.29)	
Educational attainment.			0.042
Illiteracy	137 (17.65%)	1.0	
Primary school	273 (35.18%)	0.60 (0.40, 0.91)	
Middle school	264 (34.02%)	0.52 (0.34, 0.79)	
High school	91 (11.73%)	0.61 (0.36, 1.05)	
College or above	11 (1.42%)	0.90 (0.26, 3.08)	

Abbreviations: *MetS* Metabolic syndrome, *WC* Waist circumference, *BMI* Bodymass index, *FPG* Fasting plasma glucose, *TG* Triglyceride, *HDL-C* High density lipoprotein cholesterol, *SBP* Systolic blood pressure, *DBP* Diastolic blood pressure, *FDR* First-degree relatives of patients with diabete, *OR* Odds ratio, *SD* Standard deviation

Multivariate logistic regression analysis was utilized to evaluate the association between parity and MetS as well as its components. Compared to the parity 1 group, the ORs (95% CI) of the parity 2, 3, and ≥4 groups were 1.86 (1.25, 2.77), 3.01 (1.96, 4.62) and 3.19 (1.92, 5.29), respectively, the P for trend < 0.001(Table 3, Model 1), and after adjusting for age, BMI, education level, first-degree relatives of patients with diabetes (FDR), smoking status, alcohol drinking status, physical activity, pregnancy losses, age at menarche, duration of reproductive years, this association was attenuated, the OR (95% CI) of the parity 2 group was 1.40 (0.89, 2.20), while those of the parity 3 and ≥4 groups were 2.00 (1.16, 3.44) and 1.87 (0.96, 3.62), respectively, with a P for trend = 0.025 (Table 3, Model 3). Further more, parity was failed to show a significantly relationship with MetS in normal-weight women when potential confounding factors including hip circumference were adjusted (P for trend was 0.054, Table 3, Model 4).

**Table 3:** Relationship between parity and MetS in different models in normal-weight postmenopausal women

Variable	Parity				
	1	2	3	≥4	*P* for trend
	OR (95%CI)	OR (95%CI)	OR (95%CI)	OR (95%CI)	
Crude	1	1.86 (1.25, 2.77)	3.01(1.96, 4.62)	3.19 (1.92, 5.29)	< 0.001
Model 1	1	1.51 (0.99, 2.30)	2.12 (1.30, 3.46)	2.10 (1.18, 3.75)	0.004
Model 2	1	1.52(0.98, 2.37)	2.14(1.26, 3.64)	2.13(1.13, 4.01)	0.009
Model 3	1	1.40 (0.89, 2.20)	2.00 (1.16, 3.44)	1.87 (0.96, 3.62)	0.025
Model 4	1	1.37 (0.86,2.16)	1.91 (1.10, 3.32)	1.70 (0.87, 3.33)	0.054

Model 1: adjusted for age;

Model2: adjusted for model 1 +smoking, drinking, exercise, education first-degree relatives of patients with diabetes, pregnancy losses, age at menarche, duration of reproductive years, exercise;

Model3: adjust for model 2+BMI;

Model4: adjust for model 3+hip circumference;

Abbreviations: *CI* Confidence interval, *OR* Odds ratio, *MetS* Metabolic syndrome

Among the components of MetS, women with a higher parity showed a significantly higher prevalence of elevated WC than those in the parity 1 group even after confounding factors, such as age, BMI, education level, FDR, smoking status, alcohol drinking status, physical activity, pregnancy losses, age at menarche, and duration of reproductive years, hip circumference, were further adjusted [adjusted OR (95%CI) for the parity 3 group was 2.06(1.13, 3.77), while that of the parity ≥ 4 group was 3.08(1.42, 6.66), respectively, P for trend was 0.002, Table 4 Model 3]. No significant difference was observed for the other components of MetS among the different parity groups (*P* > 0.05) (Table 4).

**Table 4:** Association between parity and the prevalence of metabolic disorders

Parity	Crude		Model 1		Model 2		Model 3	
	OR(95%CI)	*P*-value	OR (95%CI)	*P*-value	OR (95%CI)	*P*-value	OR (95%CI)	*P*-value
Abdominal obesity								
1	Refrence		Refrence		Refrence		Refrence	
2	1.58 (1.11, 2.27)	0.012	1.68 (1.14, 2.46)	0.009	1.45 (0.93, 2.24)	0.099	**1.33 (0.83, 2.14)**	**0.234**
3	2.57 (1.71, 3.87)	< 0.001	2.83 (1.76, 4.54)	< 0.001	2.54 (1.46, 4.40)	0.001	**2.06 (1.13, 3.77)**	**0.019**
≥4	4.46 (2.61, 7.61)	< 0.001	4.99 (2.72, 9.17)	< 0.001	4.25 (2.11, 8.56)	< 0.001	**3.08 (1.42, 6.66)**	**0.004**
P for trend	1.63 (1.40, 1.90)	< 0.001	1.70 (1.41, 2.05)	< 0.001	1.63 (1.31, 2.02)	< 0.001	**1.46 (1.15, 1.86)**	**0.002**
High FPG								
1	Refrence		Refrence				Refrence	
2	1.32 (0.90, 1.92)	0.153	1.20 (0.80, 1.79)	0.385	1.15 (0.75, 1.76)	0.516	1.18 (0.77, 1.81)	0.460
3	1.54 (1.01, 2.33)	0.043	1.31 (0.81, 2.11)	0.276	1.22 (0.72, 2.07)	0.450	1.27 (0.75, 2.16)	0.375
≥4	1.38 (0.83, 2.28)	0.210	1.14 (0.64, 2.03)	0.664	1.04 (0.55, 1.97)	0.911	1.10 (0.57, 2.11)	0.777
P for trend	1.14 (0.98, 1.33)	0.081	1.06 (0.89, 1.27)	0.531	1.03 (0.84, 1.25)	0.806	1.05 (0.85, 1.28)	0.672
High Bp								
1	Refrence		Refrence				Refrence	
2	1.46 (1.02, 2.08)	0.040	1.16 (0.79, 1.70)	0.460	1.01 (0.67, 1.52)	0.966	1.02 (0.67, 1.53)	0.942
3	2.13 (1.42, 3.18)	< 0.001	1.45 (0.91, 2.30)	0.117	1.16 (0.70, 1.93)	0.559	1.20 (0.72, 2.00)	0.489
≥4	2.60 (1.58, 4.26)	< 0.001	1.65 (0.94, 2.90)	0.084	1.27 (0.68,2.37)	0.461	1.31 (0.69, 2.49)	0.401
P for trend	1.40 (1.21, 1.62)	< 0.001	1.19 (1.00, 1.42)	0.049	1.09 (0.89, 1.33)	0.395	1.10 (0.90, 1.35)	0.332
Elevated TG								
1	Refrence		Refrence				Refrence	
2	1.49 (1.04, 2.14)	0.031	1.06 (0.72, 1.57)	0.755	1.03 (0.68, 1.57)	0.873	1.00 (0.66, 1.52)	0.990
3	1.48 (0.99, 2.22)	0.054	0.84 (0.52, 1.34)	0.463	0.88 (0.53, 1.47)	0.619	0.83 (0.49, 1.40)	0.482
≥4	1.34 (0.82, 2.17)	0.242	0.67 (0.38, 1.19)	0.175	0.71 (0.38, 1.34)	0.294	0.65 (0.34, 1.24)	0.191
P for trend	1.12 (0.97, 1.29)	0.134	0.87(0.73, 1.04)	0.126	0.89 (0.73, 1.09)	0.264	0.87 (0.71, 1.06)	0.169
low HDL-C								
1	Refrence		Refrence				Refrence	
2	1.64 (1.08, 2.49)	0.019	1.48 (0.95, 2.30)	0.083	1.41 (0.88, 2.26)	0.156	1.37 (0.85, 2.21)	0.190
3	1.48 (0.93, 2.35)	0.096	1.24 (0.73, 2.11)	0.422	1.13 (0.63, 2.01)	0.690	1.10 (0.61, 1.97)	0.756
≥4	1.47 (0.85, 2.56)	0.168	1.20 (0.64, 2.25)	0.577	1.01 (0.50, 2.05)	0.978	0.96 (0.47, 1.97)	0.910
P for trend	1.12 (0.96, 1.32)	0.152	1.03 (0.85, 1.25)	0.745	0.98(0.78, 1.22)	0.831	0.96 (0.77, 1.20)	0.741

Model 1: adjusted for age;

Model2: adjusted for model 1 + BMI, smoking, drinking, exercise, education first-degree relatives of patients with diabetes, pregnancy losses, age at menarche, duration of reproductive years, exercise;

Model3: adjust for model 2 +hip circumference;

Abbreviations: *CI* Confidence interval, *OR* Odds ratio, *FPG* Fasting plasma glucose, *TG* Triglyceride, *HDL-C* High density lipoprotein cholesterol, *Bp* Blood pressure

## Discussion

To the best of our knowledge, few studies have evaluated the association between parity and MetS in normal-weight postmenopausal women.

Several cross-sectional studies have concluded that parity was independently associated with an increased prevalence of MetS in different races and ethnicities [20–24]. Young Lee et al. conducted a cross-sectional study on 4098 Korean postmenopausal women and reported according to 5 groups of parity (0, 1, 2, 3 and ≥4). Accordingly, only higher parity (≥ 3 live births) was significantly associated with MetS when the parity 2 group was taken as reference (parity 3: OR 1.40 and ≥4: OR 1.38]) [20]. Ortiz et al. found that women with MetS were also more likely to have had at least three children (*P*=0.05) [25]. In addition, findings from a U.S. Hispanic/Latina study have shown that compared to one birth, those with four births had the highest odds of overall MetS (OR=1.4, 95%CI 1.0, 2.0) after adjusting for confounding factors including education, marital status, income, nativity, smoking, physical activity, menopausal status, oral contraceptive use, and hormone therapy [23]. However, Shamima Akter concluded that only pre-menopausal women with the highest parity (≥ 4) had 1.65 times higher odds of having MetS compared to those in the lowest parity (0–1), but not among postmenopausal women [22]. All of the aforementioned studies were conducted using general MetS, and none of these studies have focused on the relationship between parity and MetS in normal weight individuals. Specifically, only YAO et al. has compared the associations between parity and MetS as well as its components in two groups according to BMI (normal weight vs overweight) [26]. Here, they concluded that there was a significant statistical difference between normal weight (BMI ≤ 25 kg/m^2^) and higher weight (BMI >25kg/m^2^) groups in terms of associations between parity and MetS (P-interaction< 0.001) [26]. However, they did not further evaluate the associations between parity and MetS as well as its components in the normal weight group. The results of our study was not in accordance with the results described above. We believed that only normal weight subjects were enrolled in our research might account for the inconsistent results. Moreover, when hip circumference was excluded in confounding factors, higher parity was found to be significantly associated with higher risk of MetS. The protective effect of large hip circumference [27] have been confirmed in previous study, and it was found that a lower gluteofemoral fat mass was associated with risk of cardiovascular disease in women who were post-menopausal and had a normal BMI [28]. In present study, women with higher parity have a larger hip circumference which could explain part of attenuation of association between parity and MetS after adjusting for hip circumference.

By further analyzing the components of MetS, no consensus was reached regarding the association between parity and WC as well as other metabolic disorders. Blaudeau TE conducted a cross-sectional study in order to assess total body fat and intraabdominal adiposity in 170 nonsmoking Caucasian women and found no relationship between parity and waist circumference (*P*= 0.16) [29]. Moreover, parity was also found to not be associated with central obesity (wc=88 cm) both in an unadjusted and multivariable adjusted model (*p*= 0.66) [22]. Koch et al. found that parity was associated with BMI, but was not related to WC [30]. However, A.A. Mansour et al. concluded that the number of births remained significantly and independently associated with increased WC after adjusting for age, BMI, employment, education, and marital status [OR=1.10, 95%CI(1.06,1.12)] [31]. In addition, researchers also found that parity was significantly associated with risk of abdominal obesity measurements (WC), exhibiting a greater OR than general obesity measurements (BMI) [32]. The present results demonstrated that high parity was found to be associated with increased WC. Potential reasons pertaining to the disparate results from this study could be due to the heterogeneity in the background of the study populations. Moreover, our study was conducted in women of normal weight, thus, it may be plausible that WC may play an important role in the relationship of parity and MetS especially in the normal weight individuals.

Potential biological mechanisms may explain the association between parity and WC, Excessive fat accumulation and postpartum weight retention induced by excess calories taken during pregnancy play a role in the pathophysiological mechanism of abdominal obesity. Studies have suggested that parity may have some effect on fat distribution and intra-abdominal adipose tissue increases with increasing parity [29], which has larger influences on central obesity than on overall obesity [24]. Furthermore, insulin resistance triggered by hormonal changes during pregnancy and relative increments in insulin may promote lipid synthesis [33] and triacylglycerol surplus to deposit as visceral adipose tissue [34], whereas repeated pregnancies may amplify such effect. In addition, the release of placental corticotropin-releasing hormone during pregnancy may result in excess cortisol exposure, causing intra-abdominal adipose tissue accumulation [35]. The last but not least, each pregnancy leads to a reduced lifetime exposure to estrogen, considered that contributed to higher risk of MetS [24].

In the present study, the association between parity and other metabolic components (high fasting blood glucose, high blood pressure, elevated triglycerides and low HDL cholesterol) were not detected. The aforementioned studies have previously reported inconsistent results regarding the relationship between parity and other metabolic components. In this regard, it is believed that the socio-economic status of the study populations as well as different correction factors may partially account for the discrepancy.

We came to the conclusion that parity was not associated with MetS and associated with WC in normal weight individuals after adjusting for demographic, lifestyle factors, specially hip circumference. The effects of multiparity on fat distribution could explain the results. However, various limitations exist in the present study. First, recall bias was inevitable as information about reproductive factors was collected based on electronic medical records. Second, variables of pregnancy related complications, such as gestational diabetes and pregnancy induced hypertension, were not taken into account in the data analysis as most postmenopausal women were not even aware of the related history of pregnancy complications. Third, we take consideration of hip circumference in analyzing the association between parity and MetS, as well as WC, fat distribution such as visceral fat mass, which were measured using dual-energy x-ray absorptiometry (DEXA) or magnetic resonance (MRI) were unavailable in our study, although WC is being widely used to estimate visceral fat mass. In terms of the strengths of this study, this is first study that validates the association between parity and normal weight MetS in Chinese postmenopausal women. In addition, researchers have previously reported that pregnancy loss was associated with MetS [36] and other metabolic disorders such as diabetes or nonalcoholic fatty liver disease (NALFD) [37]. In this study, reproductive variables including duration of reproductive years and pregnancy loss were adjusted as confounding factors when exploring this association, whereas pregnancy loss was ignored in most previous studies.

## Conclusions

In conclusion, there’s no correlation between parity and MetS in postmenopausal women of normal weight after controlling for confounding risk factors. As for the components of MetS, only WC was associated with multiparity even after controlling for hip circunference. It may be plausible that multiparity would likely to affect metabolic status via increased WC, whereas the effect of increased waist circumference on MetS was attenuated by accounting for hip circumference.

## Data Availability

The data used to support the findings of this study from Institutional Review Board of the second affiliated hospital and Yuying Children’s Hospital of Wenzhou Medical University but restrictions apply to the availability of these data, which were used under license for the current study, and so are not publicly available. Data are however available from the authors upon reasonable request and with permission of Institutional Review Board of the Second Affiliated Hospital and Yuying Children’s Hospital of Wenzhou Medical University.

## Abbreviations

MetS: Metabolic syndrome
BMI: Body mass index
CVD: Cardiovascular disease
WC: Waist circumference
FPG: Fasting plasma glucose
2hPG: 2 h postprandial glucose
HbA1c: Hemoglobin A1c
IQR: Interquartile ranges
SBP: Systolic blood pressure
DBP: Diastolic blood pressure
TG: Triglyceride
TC: Total cholesterol
HDL-C: High-density lipoprotein cholesterol
LDL-C: Low-density lipoprotein cholesterol
OR: Odds ratio
95% CI: 95% confidence interval
FDR: First-degree relatives of patients with diabetes
NALFD: Nonalcoholic fatty liver disease
